# Chronic exposure to bisphenol a impairs progesterone receptor-mediated signaling in the uterus during early pregnancy

**DOI:** 10.14800/rci.1369

**Published:** 2016-07-21

**Authors:** Quanxi Li, Juanmahel Davila, Milan K. Bagchi, Indrani C. Bagchi

**Affiliations:** 1 Department of Comparative Biosciences, University of Illinois at Urbana-Champaign, Urbana, Illinois 61802, USA; 2 Departments of Molecular and Integrative Physiology, University of Illinois at Urbana-Champaign, Urbana, Illinois 61802, USA

**Keywords:** BPA, Progesterone Receptor, HAND2, Uterus, Embryo implantation

## Abstract

Environmental and occupational exposure to endocrine disrupting chemicals (EDCs) is a major threat to female reproductive health. Bisphenol A (BPA), an environmental toxicant that is commonly found in polycarbonate plastics and epoxy resins, has received much attention due to its estrogenic activity and high risk of chronic exposure in human. Whereas BPA has been linked to infertility and recurrent miscarriage in women, the impact of its exposure on uterine function during early pregnancy remains unclear. In a recent publication in *Endocrinology*, we demonstrated that prolonged exposure to an environmental relevant dose of BPA disrupts progesterone receptor-regulated uterine functions, thus affecting uterine receptivity for embryo implantation and decidua morphogenesis, two critical events for establishment and maintenance of early pregnancy. In particular we reported a marked impairment of progesterone receptor (PGR) expression and its downstream effector HAND2 in the uterine stromal cells in response to chronic BPA exposure. In an earlier study we have shown that HAND2 controls embryo implantation by repressing fibroblast growth factor (FGF) expression and the MAP kinase signaling pathway, thus inhibiting epithelial proliferation. Interestingly we observed that downregulation of PGR and HAND2 expression in uterine stroma upon BPA exposure was associated with an enhanced activation of FGFR and MAPK signaling, aberrant proliferation, and lack of uterine receptivity in the epithelium. In addition, the proliferation and differentiation of endometrial stromal cells to decidual cells, an event critical for the maintenance of early pregnancy, was severely compromised in response to BPA. This research highlight will provide an overview of our findings and discuss the potential mechanisms by which chronic BPA impairs PGR-HAND2 pathway and adversely affects implantation and the establishment of pregnancy.

## Roles of estrogen and progesterone in regulation of uterine function during early pregnancy

**Figure 1:**
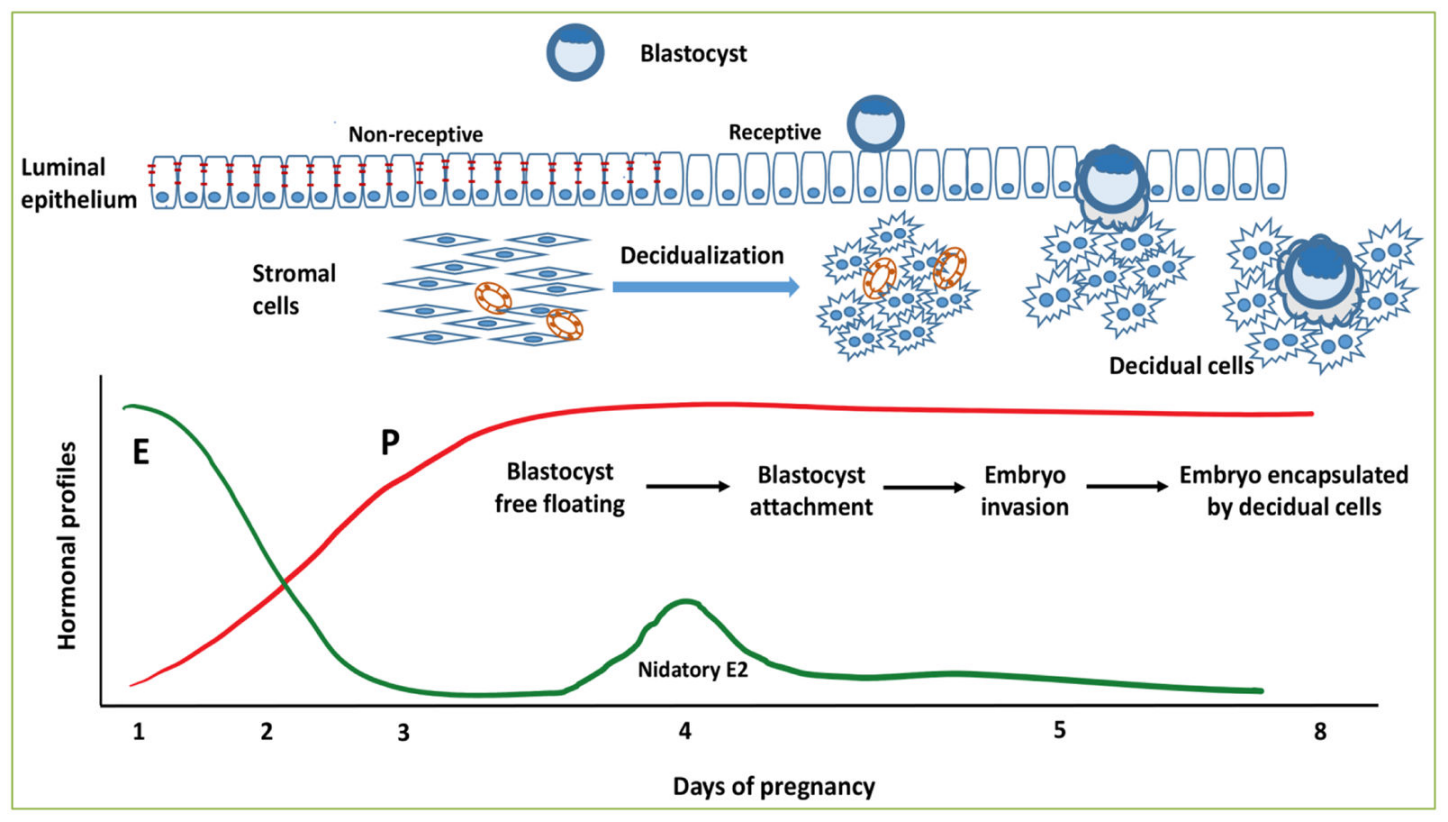
E and P-regulated events critical for establishment of early pregnancy In mice, the preovulatory ovarian E stimulates uterine epithelial proliferation on days 1 and 2 of pregnancy. Starting on day 3 of pregnancy, in response to rising P levels, epithelial cells cease to proliferate and enter a receptive differentiation program. On day 4 of pregnancy following a nidatory surge of E, uterine epithelial cells lose their polarity and allow attachment of embryo. The attached embryo breaches the epithelium barrier and triggers the P-dominated differentiation program of the subjacent fibroblastic stromal cells into secretory decidual cells that maintains an environment conducive to the growth and development of the implanting embryo. Reprinted with permission ^[51]^

The physiological functions of mammalian uterus are governed by the concerted actions of steroid hormones 17β-estradiol (E) and progesterone (P). These hormones act via their cognate receptors to control proliferation and differentiation of the endometrial uterine cells and make the uterus competent for establishment of early pregnancy. Specifically, the postovulatory E promotes growth of uterine lining by stimulating proliferation of epithelial cells. In response to rising level of P that is secreted from the newly formed corpus luteum, the uterine epithelium ceases to proliferate and undergoes differentiation to facilitate embryo attachment and invasion ^[1–3]^. This is followed by proliferation and differentiation of the underlying stromal cells ^[2, 3].^ These cells undergo morphological changes from fibroblastic to epithelioid and develop into a unique tissue, termed as decidua, which maintains an environment conducive to the growth and development of the implanting embryo (Figure 1). Thus, proper decidual morphogenesis is a prerequisite for successful implantation and establishment of pregnancy ^[1–3]^.

In the mouse, the experimentally induced implantation model (delayed implantation) provided the evidence that nidatory E on day 4 of pregnancy plays an essential role in embryo implantation and establishment of pregnancy ^[4]^. In this model, deprivation of endogenous steroid hormones by removal of ovaries prior to embryo attachment leads to suspension of embryo implantation. Administration of P to these animals allows the embryos to be viable at the blastocyst stage, but is insufficient to initiate the implantation process. However, administration of E to these P-primed pregnant mice allows attachment of the blastocyst to the luminal epithelium within 12 to 24 hours, and promotes differentiation of the underlying stromal cells to decidual cells within 48 hours. Previous studies using this model have shown that the optimal E levels for embryo implantation fall in a narrow range of 0.12 to 4 μg/kg body weight. Beyond this range, E is either insufficient or detrimental to establishment and maintenance of early pregnancy ^[4,5]^. Collectively, it is clear that uterine epithelial receptivity and stromal cell decidualization are acutely dependent on the steroid hormone signaling pathways that operate in the uterus during early pregnancy. Indeed, a slight perturbation in estrogen receptor (ESR) or progesterone receptor (PGR)-mediated signaling in the uterus leads to the development of various reproductive disorders ^[1, 2, 6]^.

## ESR1- and PGR-regulated stromal factors control uterine receptivity and stromal cell decidualization

The physiological effects of E and P are mediated by their cognate nuclear receptors. There are two different forms of the estrogen receptors, formerly referred to as ERα and ERβ, and encoded by two separate genes, *Esr1* and *Esr2*, respectively. Early studies have shown that E via ESR1 stimulates production of paracrine factors, such as IGF1 and FGFs, in uterine stromal cells. These growth factors then act on luminal epithelium and control epithelial proliferation ^[7, 8]^. There are two isoforms of PGR, PGR-A and PGR-B, generated from a single gene via different promoter usage ^[9, 10]^. Both isoforms have the same DNA binding and ligand binding domains, but PGR-B possesses an additional transactivation domain in the amino terminal region. In the presence of P, PGR dissociates from the heat shock chaperone proteins, undergoes dimerization, and binds to target genes via direct interaction with discrete DNA response elements or via tethering interactions with other transcription factors. Female mice lacking both PGR-A and PGR-B exhibit hyperplastic uteri that are non-receptive to embryo implantation and impairment in decidualization ^[11]^.

**Figure 2:**
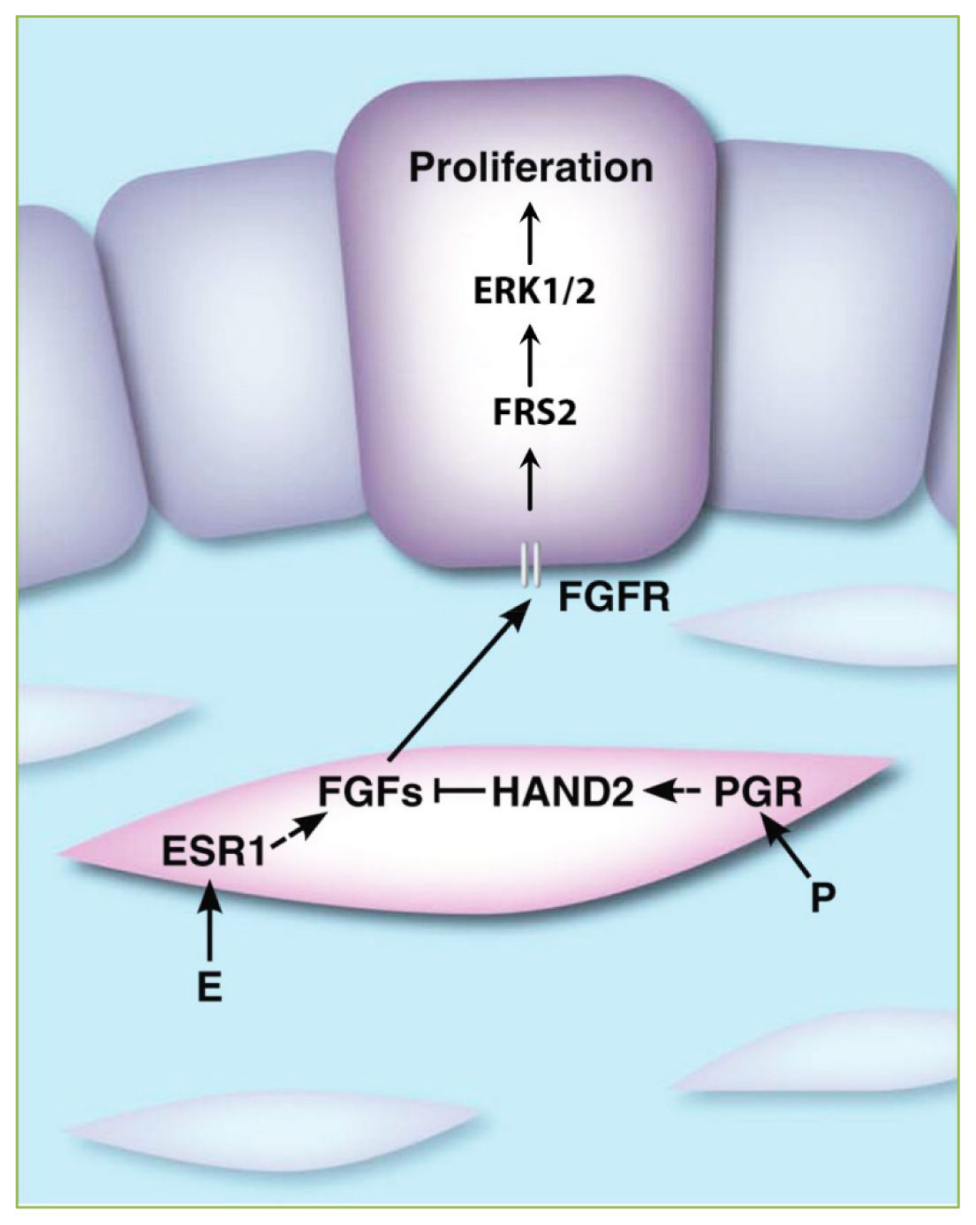
Steroid hormones regulate epithelial-stromal cross talk in the uterus during early pregnancy Before implantation, E acting through ESR1, promotes the secretion of FGFs in the fibroblastic stromal cells (pink). These growth factors in a paracrine manner act on FGF receptors in epithelial cells (purple) to activate FRS2-ERK1/2-mediated signaling pathways and drive epithelial proliferation. During the peri-implantation period, P acting on PGR in the stroma, stimulates expression of HAND2, which inhibits expression of FGFs and consequently blocks E-induced FGFR-ERK1/2-mediated MAPK signaling and inhibits epithelial proliferation. Reprinted with permission ^[1]^.

Using genome-wide gene expression profiling, we and others have identified a number of PGR-targets in the mouse and human endometrium ^[12–14]^. Our recent study showed that heart and neural crest derivatives expressed protein 2 (HAND2), a basic helix-loop-helix transcription factor, is a direct target of PGR ^[8]^. In mouse and human endometrium, *Hand2* is exclusively induced in the uterine stroma in response to P stimulation. Interestingly, our studies further revealed that stromal HAND2 plays a central role in controlling the paracrine mechanisms that mediate the anti-proliferative effects of P in the luminal epithelium. Conditional ablation of *Hand2* expression in the mouse uterus leads to infertility, primarily due to failure in embryo implantation. Further analysis of *Hand2*-null uteri revealed persistent luminal epithelial proliferation at the time of implantation, indicating that in the absence of HAND2, pregnant uteri fail to achieve the receptive status ^[8]^. We also found that HAND2 suppresses expression of a subset of stromal fibroblast growth factors (FGFs), e.g., FGF1, FGF2, FGF9, and FGF18. In the absence of HAND2, FGFs in the stroma, presumably induced by E via stromal ESR1, act in a paracrine fashion to control epithelial proliferation through activation of FGFR-FRS2-ERK1/2 pathway (Figure 2). This is also accompanied by a sustained phosphorylation and activation of epithelial ESR1 in the uterine epithelium of *Hand2*-null mice. Taken together, these findings suggest that the loss of HAND2 in the stroma prevents the epithelium to shift from a proliferative to a differentiated state, which is necessary for acquisition of receptivity for implantation.

P, acting via PGR, is the primary driver of endometrial stromal cell differentiation process ^[2, 15]^. Interestingly *Hand2* expression persists through stromal cell decidualization during early pregnancy in the mouse. Our recent studies revealed that Hand2 is a critical mediator of PGR in regulation of stromal cell differentiation (unpublished data). *Hand2*-null uterine stromal cells fail to undergo morphological transition to decidual cells and express biomarkers that are indicative of decidual uterus. In addition, HAND2 is also critical for human endometrial stromal cell decidualization. Silencing of *HAND2* expression in human endometrial stromal cells results in a decline in the secretion of decidual biomarkers, such as IGFBP1 and PRL ^[16, 17]^.

## Bisphenol A (BPA) is an environmental reproductive toxicant

Environmental and occupational exposure to BPA, an environmental endocrine disrupting chemical that is commonly found in polycarbonate plastics and epoxy resins, has received much attention in female reproductive health, primarily due to its widespread use and high risk of chronic exposure in human ^[18]^. BPA is detectable in body fluids of humans worldwide, with higher levels present in preschool children, adolescents, and occupational workers ^[19]^. Clinically, blood BPA concentrations in women are associated with reproductive disorders, such as endometrial hyperplasia, endometriosis, recurrent miscarriages, and decreased rate of pregnancy in those who seek assisted reproductive technologies (ART) ^[20–22]^. It has been reported that the human daily intake (μg/kg/day) of this chemical varies from 0.043–14.7 in children, 0.008–1.5 in adults, and 0.0043–100 in occupational workers. The level of the biologically active, unconjugated BPA measured in human serum falls in a range of 0.5–10 ng/mL, with an average level of approximately 2 ng/mL ^[18]^. Previous pharmacokinetic studies of BPA in adult CD1 female mice have shown that the bioactive unconjugated serum BPA level reaches the maximum at 1 hour (3.28 ng/mL) and the average AUC_0–24_ is 0.7 ng/mL after oral administration of 400 μg/kg BPA ^[23]^.

Early studies indicate that BPA exerts a magnitude of actions in diverse target tissues. BPA was originally identified as an estrogen mimic due to its low weak binding affinity to ESR1 and ESR2 ^[24, 25]^. Recent studies have addressed the estrogenic activity of BPA in target tissues at no or low E background. There is also increasing evidence to suggest that BPA may exhibit anti-estrogenic activities in the presence of E ^[25, 26]^. BPA also binds to the estrogen receptor-related proteins, GPR30, or estrogen receptor-related receptor γ, which are known to stimulate rapid intracellular responses through non-genomic signaling pathways ^[25, 27]^. More recent studies have also suggested that BPA exposure could lead to a long-term change in the expression levels of target genes via epigenetic mechanisms ^[28–32]^.

It is known that fetal or neonatal female rodents upon prolonged exposures to BPA encounter numerous reproductive disorders later in life, including abnormal puberty, oocyte aneuploidy, as well as a decline in reproductive capacity ^[33–38]^. The impact of the BPA exposure at environmentally relevant levels on the E and P-regulated uterine functions during early pregnancy remains unclear. In our recent publication, we investigated how chronic exposure to low levels of BPA in young female mice affects uterine epithelial receptivity and stromal cell decidualization, two critical biological events that are acutely dependent on steroid hormone-dependent signaling during early pregnancy ^[39]^.

## Chronic BPA exposure affects embryo implantation and decidualization during early pregnancy

Humans are chronically exposed to BPA primarily through oral intakes, multiple times a day ^[40]^. In order to recapitulate the BPA exposure situation in human population, particularly in teenagers, we designed a multiple dosing regimen for BPA in which young female mice were exposed to 0, 60, 600 μg/kg/day of BPA daily in three equal feedings during pubertal development. The 60 μg/kg/day dose is relevant to the average level of exposure in occupational workers and is close to the dosage of BPA that is considered safe for human consumption, which is 50 μg/kg/day ^[19]^. To circumvent the possibility that BPA exposure may impair ovarian steroidogenesis and consequently affect uterine functions, we employed the delayed model in which embryo implantation is controlled by administration of exogenous E and P. Experimental and control pregnant female mice were subjected to delayed implantation as described before. Embryo implantation and decidual response were then evaluated in these mice by 12 or 48 hours after E administration, respectively. As expected, the control pregnant females mice dosed with vehicle displayed well-formed implantation sites along the entire uterine horn. However, the females that were exposed to a chronic BPA regimen, exhibited a dose-dependent decline in the number of implantation sites. Histological analyses revealed that BPA-exposed uteri exhibited a marked impairment in decidualization as indicated by the reduction in the size of the implantation chambers. Taken together, these results indicate that chronic exposure to BPA results in an intrauterine environment that is unfavorable for embryo implantation and stromal cell decidualization during early pregnancy.

## BPA affects ESR1- and PGR-dependent molecular pathways to influence epithelial receptivity during embryo implantation

To gain insights into the mechanism by which chronic BPA exposure disrupts uterine function during early pregnancy, we first examined the expression of ESR1 and PGR in vehicle- or BPA-exposed uterine samples collected 12 hours after E administration. This period corresponds to the time of blastocyst attachment to the receptive luminal epithelium in mice. Immunohistochemistry and gene expression analyses showed that there was no appreciable difference in ESR1 expression in the uterine tissues that were dosed with or without BPA. While PGR expression in the epithelium was low but comparable in both control and BPA-exposed uterine tissues, the expression of PGR in the stroma was significantly reduced in uteri of mice that have been exposed to BPA. Consistent with these observations we found that the targets of PGR in epithelial cells, such as *Ihh*, *Alox15*, and *Irg1*, were expressed at comparable levels in vehicle- or BPA-treated uterine samples. In contrast, the stromal targets of PGR including *Hand2* and *Hoxa10* were markedly reduced in BPA-exposed uteri.

To investigate the possibility that BPA interacts with ESR1 to modulate its transcriptional activity, we monitored the effect of BPA on factors that are regulated by ESR1 and play a critical role in epithelial receptivity and stromal cell differentiation during early pregnancy. Targets of ESR1 in the luminal epithelium (*Muc1*), the glandular epithelium (*Lif*), as well as in the stromal cells (*Fra1* and *Gja1*) were selected for the analyses. Interestingly we observed that compared to the vehicle-treated controls, *Muc1* expression was significantly upregulated while expressions of *Lif*, *Fra1*, and *Gja1* were markedly downregulated in BPA-exposed uteri. Collectively these results indicate that BPA selectively interferes with ESR1- and PGR-mediated signaling pathways in the uterus during early pregnancy.

**Figure 3:**
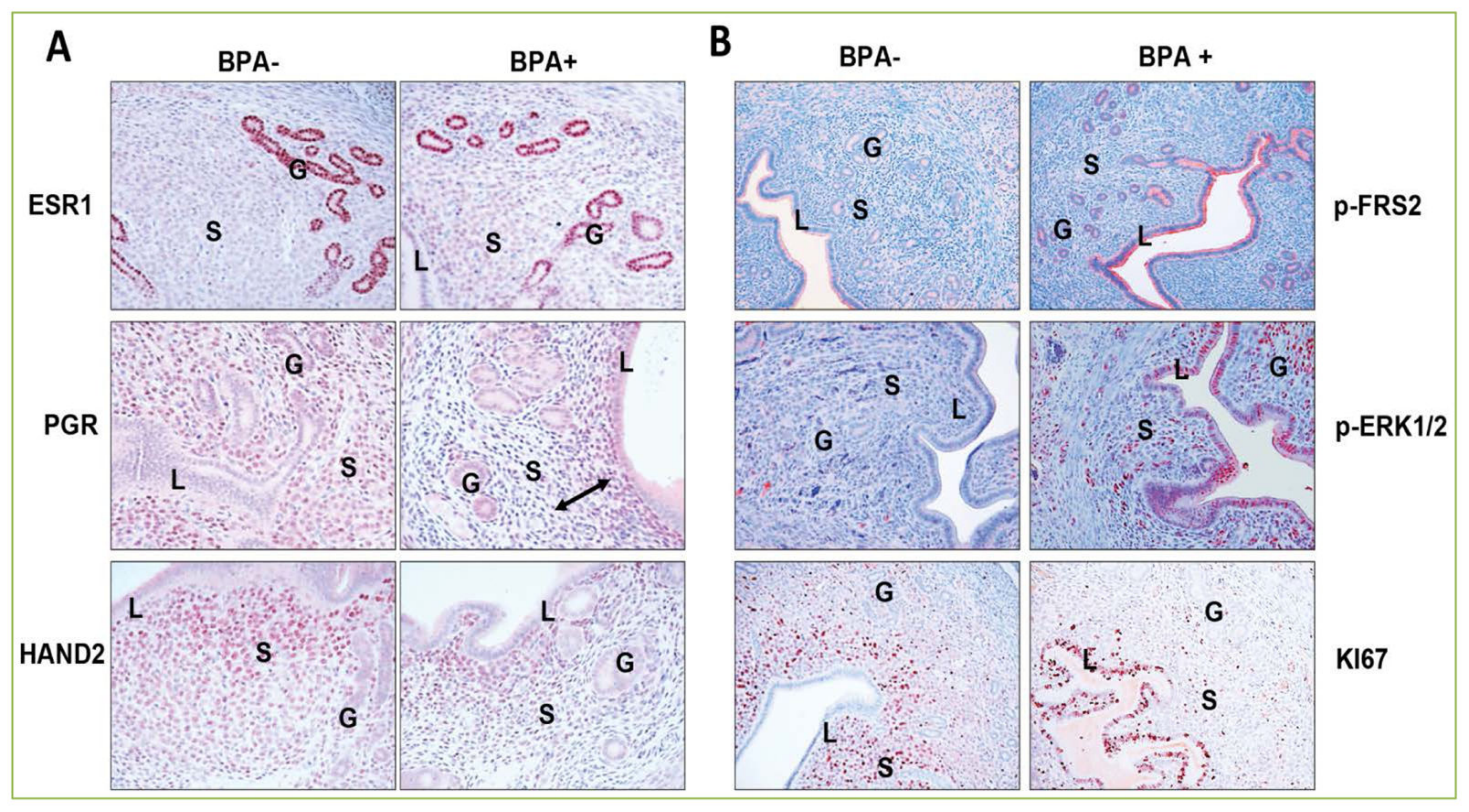
Chronic BPA exposure disrupts PGR-mediated signaling in the uterus In comparison to non-treated uterine tissues, PGR and HAND2 expression is markedly downregulated upon BPA exposure (A). Disruption of PGR-mediated pathways in BPA-exposed uterine stromal cells leads to a marked enhancement in the FGFR-ERK1/2 MAPK signaling in the epithelium and drives persistent proliferation in these cells (B). L, S, and G denote luminal epithelium, glandular epithelium, and stroma, respectively.

In many species including mice, the receptive state is marked by a cessation in epithelial cell proliferation prior to implantation. Our recent studies have shown that the cessation of epithelial proliferation is mediated by stromal Hand2, which is expressed downstream of stromal PGR. As described previously, in mouse uteri lacking *Hand2*, persistent induction of FGFs in the stroma activates FGF receptor (FGFR) and ERK1/2-mediated MAPK signaling in the epithelium to promote cell proliferation and impair implantation ^[8]^. Hence down regulation of PGR and HAND2 in the uterine stroma by BPA exposure prompted us to determine the effect of this EDC on FGFR signaling at the time of implantation. We examined the tyrosine phosphorylation status of FRS2, an adapter protein that links activated FGR receptors to downstream signaling pathways, such as ERK1/2. As expected, only low levels of phospho-FRS2 (p-FRS2) and phospho-ERK1/2 (p-ERK1/2) were observed in the uterine epithelia of control mice at the time of implantation. In contrast, a marked increase in the phosphorylation level of these two proteins was seen in the epithelia of BPA-exposed uteri, indicating elevated FGFR signaling in response to this EDC (Figure 3). Consistent with this observation we found an enhanced proliferation of uterine epithelial cells, as indicated by KI67 staining, in response to BPA exposure. We also determined the gene expression levels of FGF family members in the uterine stroma of these females and found that *Fgf1*, *Fgf7*, *Fgf9* and *Fgf18* levels are markedly elevated in BPA-exposed uteri compared to the vehicle-treated controls. These results indicate that downregulation of Hand2 in the uterine stroma in response to BPA results in persistent activation of FGFR-ERK1/2 pathway and enhanced cell proliferation in the epithelium, making the uterus non-receptive for implantation.

## Uterine stromal cell differentiation is impaired in response to BPA exposure

We further investigated the expression level of PGR in uterine tissues collected from female mice treated with or without BPA at 48 hours after E stimulation, which overlaps with the decidual phase of pregnancy. Immunohistochemical analysis revealed that BPA exposure led to an aberrant spatial expression pattern of PGR in the endometrial stroma during decidualization. In the control vehicle-exposed uterine sections, PGR was localized to the stromal cells outside the primary decidual zone comprising of a few layers of cells in the immediate vicinity of the implanted embryo. In the BPA-exposed uterine samples the cells in the primary decidual zone expressed PGR, while the cells outside this zone were devoid of PGR expression. Interestingly, when stromal cell proliferation and differentiation were assessed by immunohistochemistry using antibodies against KI67 and decidual prolactin-related protein (PRL8A2), we observed that in control uterine tissues the stromal cells in close vicinity of the embryos were largely devoid of any KI67 staining indicating that these cells have exited the cell cycle and entered the differentiation program. Indeed these cells expressed PRL8A2, a differentiation marker of stromal cells. In contrast, the uterine sections from BPA-exposed mice exhibited wide spread staining of KI67 in the stromal cells that are in close proximity to the implanted embryo. These cells did not undergo differentiation as indicated by the lack of expression of PRL8A2. These results indicate that BPA exposure affects the differentiation of uterine stromal cells to decidual cells that is critical for the establishment of pregnancy.

## Perspectives

Several lines of evidence including our current studies suggest that PGR-HAND2-mediated molecular pathway is vulnerable to exposure of an environmental toxic chemical with estrogenic or anti-estrogenic activity, including BPA. The precise mechanism by which BPA affects PGR/HAND2 expression in the uterus remains unclear. Early studies showed that BPA is capable of binding to ESR1 and ESR2 with low affinity. It has also been reported that pre-treatment of E followed by ingestion of 50 μg/kg of BPA in female rodents reduces the bioavailability of BPA in the uterus ^[41]^. It is plausible that BPA interferes with ESR1-dependent gene expression in the uterine stroma including PGR during early pregnancy. There is also increasing evidence to suggest that chronic BPA exposure could alter gene expression in target tissues through epigenetic mechanisms, including DNA methylation in “CG” islands of target gene promoters ^[28, 29, 42]^. Whereas the effect of BPA on DNA methylation of PGR has not been reported, a recent study has shown that methylation of *Stat3* and *Fkbp5* are affected in the liver of BPA-exposed mice ^[43, 44]^. Furthermore, studies from the Taylor laboratory have shown that DNA methylation of *Hoxa10*, a known target of PGR, is affected in response to BPA exposure ^[31]^. Interestingly we noted that the expression of *Dnmt3b*, a member of the methyltransferases responsible for establishing methylation patterns ^[45]^, is markedly induced in uterine stromal cells in response to chronic low-level BPA exposure. It is possible that BPA exposure affects expression of PGR and PGR-targets by promoter DNA methylation mechanism. These studies are currently under investigation in the laboratory.

Loss of PGR expression or disruption in PGR-mediated signaling is always associated with an unopposed E action/P-resistance in the endometrium that is favorable to cell cycle progression and inflammatory reaction, leading to various female reproductive diseases ^[46–50]^. Our studies revealed that chronic exposure to low levels of BPA during pubertal development in female mice adversely affects PGR-HAND2 dependent signaling in the uterus and impact the function of this tissue later in life. Hence, deciphering the underlying mechanism of aberrant steroid receptor-dependent signaling in the uterus in response to BPA exposure will provide novel insights into the BPA-associated female reproductive disorders, such as infertility, early pregnancy loss, endometriosis, endometrial hyperplasia, and endometrial cancers.

## Abbreviation

BPA: Bisphenol A
E: 17β-estradiol
EDC: Endocrine disrupting chemical
ESR: Estrogen receptor
ERK1/2: Extracellular signal-regulated kinases 1/2
FRS2: Fibroblast growth factor substrate 2
P: Progesterone
PGR: Progesterone receptor
